# Exploring the Mechanisms of the Antioxidants BHA, BHT, and TBHQ in Hepatotoxicity, Nephrotoxicity, and Neurotoxicity from the Perspective of Network Toxicology

**DOI:** 10.3390/foods14071095

**Published:** 2025-03-21

**Authors:** Jing Ren, Ziang Li, Xiaofen Li, Lin Yang, Zhulin Bu, Yuhui Wu, Yuting Li, Shuosheng Zhang, Xianglong Meng

**Affiliations:** 1 School of Traditional Chinese Medicine and Food Engineering, Shanxi University of Chinese Medicine, Jinzhong 030619, China; renjing@sxtcm.edu.cn (J.R.); liziang20221018@163.com (Z.L.); 16636400102@163.com (X.L.); 15934482789@163.com (L.Y.); bb19050901@163.com (Z.B.); 13653657042@163.com (Y.W.); liyuting010316@163.com (Y.L.); zhangshuosheng@aliyun.com (S.Z.); 2 Shanxi Key Laboratory of Traditional Chinese Medicine Processing, Shanxi University of Chinese Medicine, Jinzhong 030619, China; 3 Key Research Laboratory of Processing and Innovation in Traditional Chinese Medicinal Materials, Shanxi University of Chinese Medicine, Jinzhong 030619, China; 4 Traditional Chinese Medicine Processing Technology Inheritance Base, Shanxi University of Chinese Medicine, Jinzhong 030619, China

**Keywords:** BHA, BHT, TBHQ, hepatotoxicity, nephrotoxicity, neurotoxicity

## Abstract

The widespread use of food additives, such as butylated hydroxyanisole (BHA), butylated hydroxytoluene (BHT), and tert-butylhydroquinone (TBHQ), has raised concerns about their potential toxicity, especially their hepatotoxicity, nephrotoxicity, and neurotoxicity. This study explores the targets and mechanisms of food additive-induced toxicity using network toxicology. Toxicity predictions of BHA, BHT, and TBHQ were performed using the ProTox-3.0, ADMETlab 3.0, and Xundrug databases, and potential targets were identified using the SwissTargetPrediction, Batman-TCM, SuperPred, and SEA databases. These were integrated with GeneCards—The Human Gene Database (GeneCards) and the Online Mendelian Inheritance in Man (OMIM) database to extract toxicity-related targets for subsequent Gene Ontology (GO) and Kyoto Encyclopedia of Genes and Genomes (KEGG) analyses. Core-acting genes were further screened through protein–protein interactions (PPIs), and molecular docking was performed to verify the binding free energy between BHA, BHT, and TBHQ and their core targets. Additionally, the mRNA-miRNA-lnRNA interaction regulatory networks of the core targets and potential carcinogenic mechanisms were analyzed. The targets of BHA, BHT, and TBHQ were as follows: ACE, HIF1A, NR1H4, NFKB1, TNF, IL6, IFNG, IL1B, and ESR1 for hepatotoxicity; APP, NFKB1, ACE, FOS, IL10, IL1B, IL6, TNF, and ALB for nephrotoxicity; and GRIN2B, IL1B, and TNF for neurotoxicity. These interactions primarily involved pathways such as interleukin-17 (IL-17) and Janus kinase-signal transducer and activator of transcription (JAK-STAT), as well as various pathways related to non-alcoholic fatty liver disease (NAFLD). This study highlights the potential toxicity of BHA, BHT, and TBHQ to the liver, kidneys, and nerves, providing insights for better safety evaluations.

## 1. Introduction

Recent advancements in the food industry have elevated the concern around food safety to a critical level, impacting both the national economy and public well-being. Food oxidation significantly contributes to deteriorations in food quality, depletions in nutrients, and the generation of harmful substances [1,2]. Antioxidants are widely used as food additives to delay oxidation and maintain the color, fragrance, taste, and nutritional value of food [3]. Butylated hydroxyanisole (BHA) and butylated hydroxytoluene (BHT) and tert-butylhydroquinone (TBHQ) are prevalent antioxidants used in high-fat foods such as oils and processed items to enhance shelf life [4,5]. The long-term administration of high doses of BHA, BHT, and TBHQ may induce hepatotoxic effects and cause structural damage to renal tubules and glomeruli, potentially leading to nephrotoxicity [4]. TBHQ significantly impairs growth, development, and embryonic tissue formation in zebrafish models [6]. BHA is linked to thyroid dysfunction, and BHT disrupts endocrine function, potentially affecting hormonal balance in biological systems [7,8]. The Codex Alimentarius Commission has established globally recognized scientific standards for the use of BHA, BHT, and TBHQ. However, their safety and potential risks remain concerning [9,10].

BHA retards the oxidation of food products mainly by trapping free radicals. Chronic high-dose exposure to BHA may exacerbate hepatic inflammation through the activation of angiotensin II, which upregulates the nuclear factor kappa-light-chain-enhancer of activated B cells (NF-κB) signaling pathway, with angiotensin-converting enzyme (ACE) partially contributing to the activation of Ang II. This mechanism further induces oxidative stress and systemic inflammation, posing a potential threat to human health. Additionally, BHA exhibits potential carcinogenic and endocrine-disrupting properties, although these risks require further comprehensive investigation [11,12,13]. Time- and dose-dependent exposure to BHA triggers apoptotic cell death in A549 cells, mediated by structural perturbations of nuclear DNA and the chromatin architecture, which ultimately lead to growth arrest. Through mitochondrial membrane permeabilization and subsequent cytochrome c efflux, BHA exposure in vitro promotes a mixed cell death phenotype characterized by concurrent apoptosis and necrosis. This dual-pathway disruption underlines its multifaceted toxicity profile, which encompasses DNA damage, cytotoxic cell clearance, and tumorigenic potential [8,10]. BHT is also a fat-soluble antioxidant and is widely used in various fatty foods and their packaging. Studies have shown that an excessive intake of BHT may have toxic effects on animal and human health, especially at high doses and with chronic exposure, which may trigger liver damage, cancer, and other chronic diseases [9,14]. BHT induces oxidative stress in murine 3T3 fibroblasts through the overproduction of intracellular reactive oxygen species (ROS), culminating in cellular damage and apoptosis, with a significant cytotoxic threshold observed at 300 μg/mL [15]. TBHQ is mainly used for the fresh-keeping of vegetable and animal oils [16]. However, chronic high doses of TBHQ may also trigger toxic reactions, such as liver damage [17,18]. In addition, TBHQ has been shown to cause potential damage to the kidney, including damage to the tubules and glomeruli, which, in turn, impacts overall renal function [19,20]. TBHQ induces an inflammatory response by activating the Kelch-like ECH-associated protein 1 (KEAP1) pathway, leading to a significant upregulation of proinflammatory cytokines, including IL1B, IL-6, and TNF-α. Moreover, TBHQ directly promotes the transcription and secretion of IL-6 through a mechanism involving EGFR phosphorylation and the sustained activation of the downstream extracellular signal-regulated kinase (ERK) signaling pathway, thereby further amplifying the inflammatory cascade [21]. The metabolic derivative of TBHQ, namely, tert-butylbenzoquinone (TBBQ), impedes DNA synthesis via S/G2 cell cycle arrest and induces oxidative DNA damage through intercalation-mediated mechanisms, as evidenced by elevated 8-hydroxy-2′-deoxyguanosine (8-OHdG) adduct formation [17]. Thus, in the aforementioned literature, BHA, BHT, and TBHQ have been demonstrated to exhibit potentially hepatotoxic, nephrotoxic, and neurotoxic effects; however, the precise mechanisms by which they exert these effects continue to be issues not elaborated on in detail at the nexus of computational toxicology, systems biology, and regulatory science.

In this study, network toxicology and bioinformatics were employed to systematically explore molecular-level interactions and the mechanisms between chemicals and biological systems. Furthermore, network toxicology and molecular docking were also employed to investigate the molecular mechanisms underlying the potential hepatotoxicity, nephrotoxicity, and neurotoxicity of BHA, BHT, and TBHQ. Potential mRNA-miRNA-lncRNA interaction networks were explored and core chromosomal genes were identified, with preliminary validation through molecular docking. The aim of this study was to offer innovative approaches for a thorough assessment of the safety of BHA, BHT, and TBHQ as food additives.

## 2. Materials and Methods

### 2.1. Preliminary Toxicity Analysis of BHA, BHT, and TBHQ

**Figure 1:**
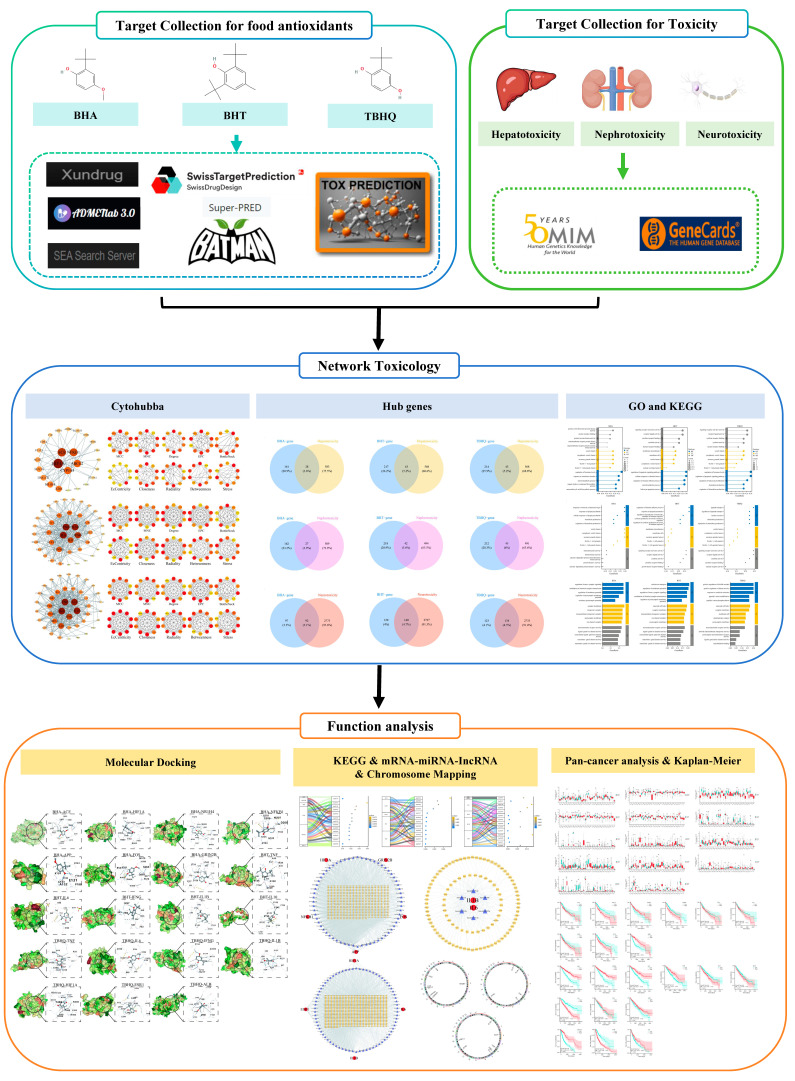
Technical roadmap.

A model was used to predict the toxicity of BHA, BHT, and TBHQ. Potential toxicity data for BHA, BHT, and TBHQ were gathered using the ProTox-3.0 [22], ADMETlab 3.0 [23], and Xundrug databases as initial screening resources. Figure 1 provides a schematic representation of the research conceptualization and technical implementation strategy employed in this study. Table 1 comprehensively presents the key databases and analytical software packages employed in the current research.

### 2.2. Target Collection for BHA, BHT, and TBHQ

The structural formulas and SMILES numbers of BHA, BHT, and TBHQ were obtained from the PubChem database. Potential targets of BHA, BHT, and TBHQ were searched for using these SMILES numbers in the SwissTargetPrediction, Batman-TCM, SuperPred, and similarity ensemble approach (SEA) databases [24], and the searched race was set to “Homo sapiens”. The resulting target UniProt IDs were entered into the UniProt database and converted into gene symbols for normalization. The identified targets were organized into potential targets of BHA, BHT, and TBHQ.

### 2.3. Target Collection for Hepatotoxicity, Nephrotoxicity, and Neurotoxicity

In the GeneCards and OMIM databases, “Hepatotoxicity”, “Nephrotoxicity”, and “Neurotoxicity” were searched. The protein-coding gene sets identified in the GeneCards database were taken and combined with the targets identified in the OMIM database, thus providing potential targets for hepatotoxicity, nephrotoxicity, and neurotoxicity.

### 2.4. Functional Enrichment Analysis

The potential targets of BHA, BHT, and TBHQ for liver, kidney, and nerve injury were analyzed via GO and KEGG pathway enrichment analyses using Xiantao Academic. A GO enrichment analysis mainly includes three aspects: a biological process (BP), molecular function (MF), and cellular component (CC). BP describes the biological activities or processes that the gene product is involved in; MF focuses on the function of the gene product at the molecular level; and CC refers to the cellular or subcellular location of the gene product. The biological significance and function of potential gene sets were revealed using the GO enrichment analysis. In addition, the KEGG enrichment analysis was performed to identify important pathways associated with the potential targets of BHA-, BHT-, and TBHQ-induced hepatotoxicity, nephrotoxicity, and neurotoxicity, and a *p*.adjust threshold of <0.05 was set to identify the main pathways of action of the obtained targets.

Further analysis of the core targets of BHA, BHT, and TBHQ involved in the hepatotoxicity, nephrotoxicity, and neurotoxicity pathways was conducted to elucidate and highlight the important signaling pathways involved in biological processes. A visualization analysis was also performed to effectively interpret and present the GO and KEGG analysis results.

### 2.5. Construction of Protein Interaction Network and Screening of Core Targets

The potential targets of BHA, BHT, and TBHQ for hepatotoxicity, nephrotoxicity, and neurotoxicity were entered into the STRING database, and the species was set to “Homo sapiens”, with a confidence > 0.4 used to identify genes with reliable interactions for a subsequent analysis of the active proteins corresponding to the target genes. Then, the results obtained from the STRING database [25] were input into Cytoscape software (version 3.9.0) [26], which visualizes and analyzes biological processes through networks, calculates the topological characteristics of the input nodes and edges, and generates a protein–protein interaction (PPI) network diagram using the node degree as a standard. In a PPI analysis, the degree value indicates the number of connections between a gene and other genes, and the higher the degree value, the stronger the connectivity of the gene in the network, thus indicating that it may play a more important role in the examined biological process. At the same time, the Cytohubba plugin was further used to cluster the input proteins from this topology to help identify potential critical genes or proteins by calculating the importance of the nodes in the network. A cluster analysis was performed to examine 10 aspects: maximum clique centrality (MCC), maximum neighborhood centrality (MNC), degree, eigenvector centrality (EC), bottleneck, eccentricity, closeness, radiality, betweenness, and stress. These aspects were used to assess the importance, centrality, and criticality of the proteins. Finally, the results of the analyses of BHA, BHT, and TBHQ in terms of these ten aspects were compared to identify the core action targets for hepatotoxicity, nephrotoxicity, and neurotoxicity.

### 2.6. Molecular Docking of BHA, BHT, and TBHQ to Core Target Proteins During Hepatotoxicity, Nephrotoxicity, and Neurotoxicity

The molecular interactions between BHA, BHT, and TBHQ and the core targets of hepatotoxicity, nephrotoxicity, and neurotoxicity were further analyzed by predicting the binding free energy using the molecular docking method. The 3D structures of BHA, BHT, and TBHQ were downloaded from the PubChem database, while the crystal structures of the core proteins were obtained from the RCSB (Research Collaboratory for Structural Bioinformatics) Protein Data Bank (PDB). The core proteins were processed using Pymol to remove their original ligands, and then molecular docking was carried out using the CB-DOCK2 database [27].

### 2.7. Functional Pathway Analysis of Core Targets 

The core targets of BHA, BHT, and TBHQ during hepatotoxicity, nephrotoxicity, and neurotoxicity were examined via a KEGG pathway enrichment analysis using Xiantao Academic. The pathways associated with hepatotoxicity, nephrotoxicity, and neurotoxicity were screened for a *p*.adjust < 0.05, and a mulberry bubble diagram was constructed to analyze the correlations of genes and pathways with the core genes of BHA, BHT, and TBHQ.

### 2.8. Construction of mRNA-miRNA-lncRNA Interaction Network of Core Targets

The miRNAs of the core targets of BHA, BHT, and TBHQ for hepatotoxicity, nephrotoxicity, and neurotoxicity, selected after six steps, were searched for in the miRDB [28], miRWalk [29], and starBase databases [30], respectively. If a target was found to have miRNAs that were common among the three, then their miRNAs were used; if not, then the target was removed. The resulting miRNAs were used to predict the IncRNAs in the starBase database and to map mRNA-miRNA-IncRNA networks in vivo, thereby allowing for the identification of the core genes of these three substances using Cytoscape software.

### 2.9. Chromosomal Localization Analysis of Core Targets

Detailed information on the sequence, structure, and functional annotation of H. sapiens genes was obtained from the Gencode database. clusterProfiler in the R language and the org.Hs.eg.db and RCircos packages were used to extract gene annotation information. The IDs of the core genes obtained were converted, and then chromosomal localization maps of the core genes of BHA, BHT, and TBHQ were drawn [31].

### 2.10. Carcinogenic Risk Analysis of Core Targets

BHA, BHT, and TBHQ were analyzed using the TCGA database, and the survival prognosis of liver cancer, renal clear cell carcinoma, and glioma was further analyzed using Xiantao Academic.

## 3. Results

### 3.1. Toxicity Prediction

The toxicity of BHA, BHT, and TBHQ was predicted using three databases. According to the ProTox3.0 database, BHA, BHT, and TBHQ exhibit grade 4 toxicity, with noted effects on the nervous system and liver. The predicted median lethal dose (LD50) of BHA, BHT, and TBHQ was 880 mg/kg, 650 mg/kg, and 700 mg/kg, respectively. A summary of the regulatory toxicological assessments of the three compounds, encompassing in silico hazard predictions and Codex Alimentarius-compliant dosage thresholds, is presented in Table 2. This lays a foundation for us to further study the toxic effects of these three compounds on humans.

### 3.2. Potential Targets of BHT, BHT, and TBHQ for Hepatotoxicity, Nephrotoxicity, and Neurotoxicity

In the search of the GeneCards and OMIM databases, 611 targets for hepatotoxicity, 563 targets for nephrotoxicity, and 2867 targets for neurotoxicity were found (Supplementary Table S1). The potential targets of BHA, BHT, and TBHQ for hepatotoxicity, nephrotoxicity, and neurotoxicity were obtained through an intersection analysis: 189 for BHA, 260 for BHT, and 257 for TBHQ (Supplementary Table S2). The potential number of targets of BHA, BHT, and TBHQ for hepatotoxicity was 28, 43, and 43, respectively (Supplementary Table S3) (Supplementary Figure S1A–C). The potential number of targets of BHA, BHT, and TBHQ for nephrotoxicity was 27, 42, and 45, respectively (Supplementary Table S4) (Supplementary Figure S1D–F). The potential number of targets of BHA, BHT, and TBHQ for neurotoxicity was 92, 140, and 134, respectively (Supplementary Table S5 and Supplementary Figure S1G–I). BHA, BHT, and TBHQ exhibited overlapping toxicological effects, particularly in terms of hepatotoxicity, nephrotoxicity, and neurotoxicity, highlighting their shared molecular targets and mechanisms of action.

### 3.3. GO and KEGG Enrichment Analyses of Potential Targets

**Figure 2:**
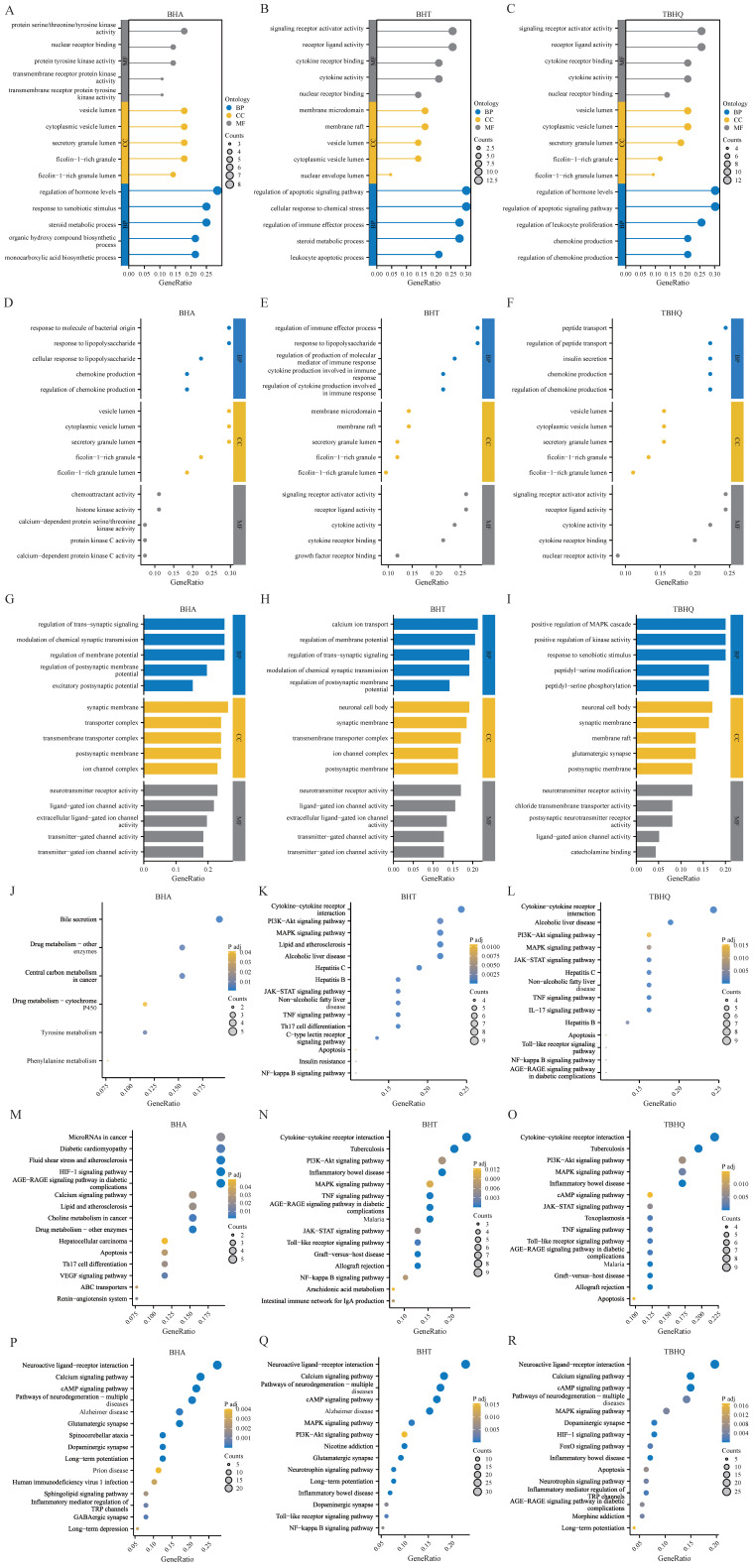
GO and KEGG enrichment analyses of targets of BHA, BHT, and TBHQ for hepatotoxicity, nephrotoxicity, and neurotoxicity. GO enrichment analysis of targets of BHA (**A**), BHT (**B**), and TBHQ (**C**) for hepatotoxicity; GO enrichment analysis of targets of BHA (**D**), BHT (**E**), and TBHQ (**F**) for nephrotoxicity; GO enrichment analysis of targets of BHA (**G**), BHT (**H**), and TBHQ (**I**) for neurotoxicity. KEGG enrichment analysis of targets of BHA (**J**), BHT (**K**), and TBHQ (**L**) for hepatotoxicity; KEGG enrichment analysis of targets of BHA (**M**), BHT (**N**), and TBHQ (**O**) for nephrotoxicity; KEGG enrichment analysis of targets of BHA (**P**), BHT (**Q**), and TBHQ (**R**) for neurotoxicity.

The GO analysis of BHA, BHT, and TBHQ showed the following: Their hepatotoxicity mainly affected biological processes, molecular functions, and cellular components such as nuclear receptor binding, protein tyrosine kinase activity, the regulation of the immune process, and the regulation of chemokine production (Figure 2A–C). Their nephrotoxicity mainly affected the response to lipopolysaccharides, protein kinase C activity, the regulation of cytokine production involved in the immune response, growth factor receptor binding and insulin secretion, and nuclear receptor activity (Figure 2D–F). Finally, their neurotoxicity mainly affected the regulation of the membrane potential, neurotransmitter receptor activity, neuronal cell bodies, glutamatergic synapses, and the positive regulation of the mitogen-activated protein kinase (MAPK) cascade (Figure 2G–I). However, the KEGG pathway analysis of BHA, BHT, and TBHQ showed the following: Their hepatotoxicity mainly involved the phosphoinositide 3-kinase/protein kinase B (PI3K-Akt) signaling pathway, IL-17 signaling pathway, apoptosis signaling pathway, drug signaling pathway-other enzymes, and nuclear factor kappa-light-chain-enhancer of activated B cells (NF-κB) signaling pathway (Figure 2J–L). Their nephrotoxicity mainly involved the MAPK signaling pathway, apoptosis, the TNF signaling pathway, the vascular endothelial growth factor (VEGF) signaling pathway, the Janus kinase-signal transducer and activator of transcription (JAK-STAT) signaling pathway, and other pathways (Figure 2M–O). Finally, their neurotoxicity mainly involved the calcium signaling pathway, Alzheimer’s disease, the NF-κB signaling pathway, long-term depression, the Forkhead Box O (FoxO) signaling pathway, and inflammatory bowel disease (Figure 2P–R).

### 3.4. Core Target Analysis

**Figure 3:**
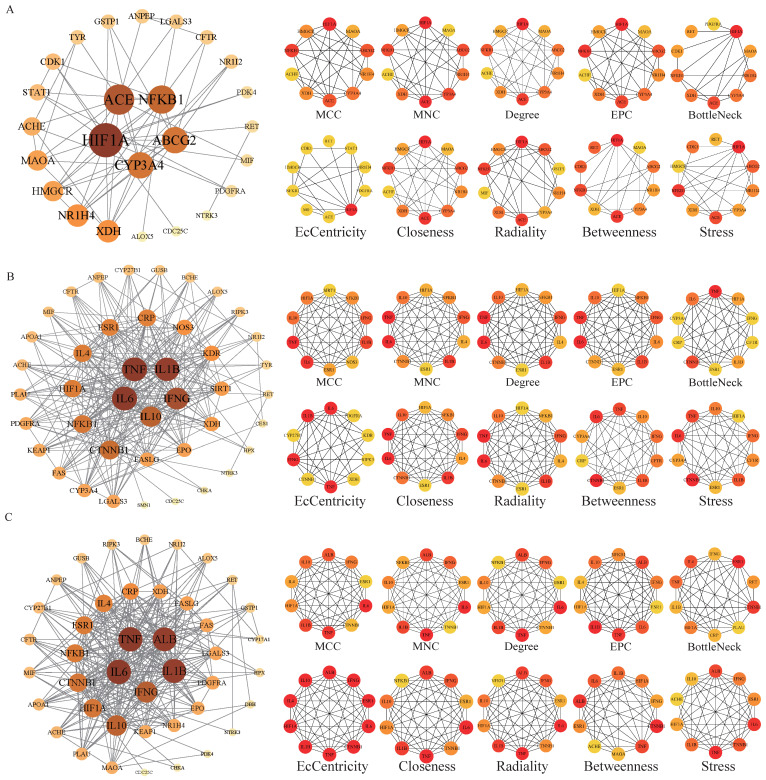
Cytohubba analysis of the core targets of BHA, BHT, and TBHQ in hepatotoxicity using Cytoscape. The hepatotoxicity core target network map and Cytohubba analysis of BHA (**A**), BHT (**B**), and TBHQ (**C**). Node color represents centrality, with dark brown indicating high centrality and light yellow indicating low centrality. The depth of color is positively correlated with centrality.

**Figure 4:**
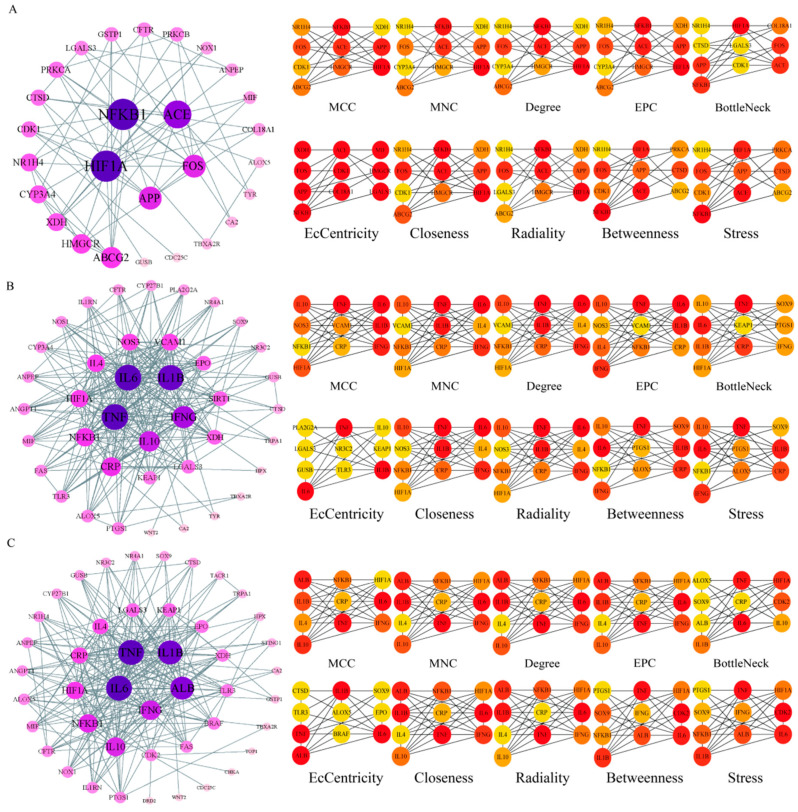
Cytohubba analysis of core targets of BHA, BHT, and TBHQ in nephrotoxicity using Cytoscape. The nephrotoxicity core target network map and Cytohubba analysis of BHA (**A**), BHT (**B**), and TBHQ (**C**). Node color represents centrality, with deep purple indicating high centrality and light pink indicating low centrality. The depth of color is positively correlated with centrality.

**Figure 5:**
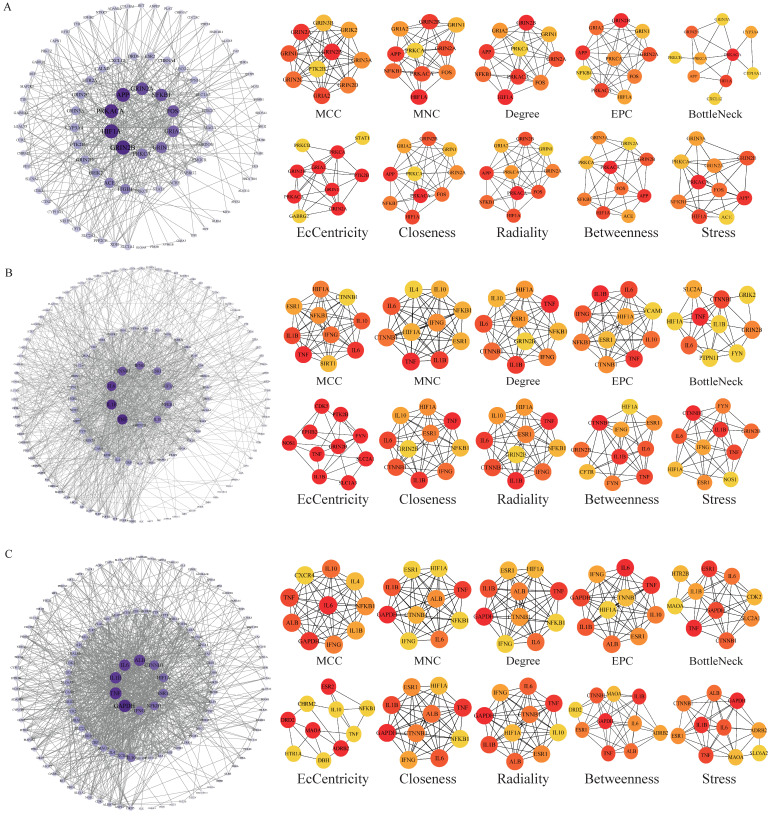
Cytohubba analysis of core targets of BHA, BHT, and TBHQ in neurotoxicity using Cytoscape. The neurotoxicity core target network map and Cytohubba analysis of BHA (**A**), BHT (**B**), and TBHQ (**C**). Node color represents centrality, with deep purple indicating high centrality and light purple indicating low centrality. The depth of color is positively correlated with centrality.

The potential targets of BHA, BHT, and TBHQ for hepatotoxicity, nephrotoxicity, and neurotoxicity were analyzed using a topological network analysis in Cytoscape and screened based on their degree value to obtain their corresponding PPI action networks. Further clustering using a Cytohubba analysis showed that the intersecting genes were the core targets of hepatotoxicity, nephrotoxicity, and neurotoxicity. Among them, the targets of BHA, BHT, and TBHQ in hepatotoxicity were ACE, HIF1A, NR1H4, NFKB1, and TNF; IL6, IFNG, IL1B, and TNF; and IL6, IFNG, IL1B, HIF1A, ESR1, respectively (Figure 3A–C). The targets of BHA, BHT, and TBHQ in nephrotoxicity were APP, NFKB1, ACE, and FOS; IL10, IL1B, IL6, and TNF; and ALB, TNF, IL6, and IL1B (Figure 4A–C). The targets of BHA, BHT, and TBHQ in neurotoxicity were GRIN2B, IL1B and TNF, and TNF, respectively (Figure 5A–C). BHT and TBHQ exhibited overlapping targets in hepatotoxicity, including IL6, TNF, IFNG, and IL1B, while HIF1A was identified as a target shared by BHA and TBHQ. In nephrotoxicity, BHT and TBHQ jointly targeted IL1B, IL6, and TNF. Similarly, TNF was a shared target of BHT and TBHQ in neurotoxicity. Furthermore, NFKB1 and ACE were common targets of BHA in liver, kidney, and nerve injury, whereas BHT and TBHQ consistently targeted IL6, TNF, and IL1B across these toxicological endpoints. These findings highlight the shared molecular mechanisms underlying the toxic effects of these compounds (Supplementary Table S6).

### 3.5. Molecular Docking of BHA, BHT, and TBHQ with Core Target Proteins in Hepatotoxicity, Nephrotoxicity, and Neurotoxicity

**Figure 6:**
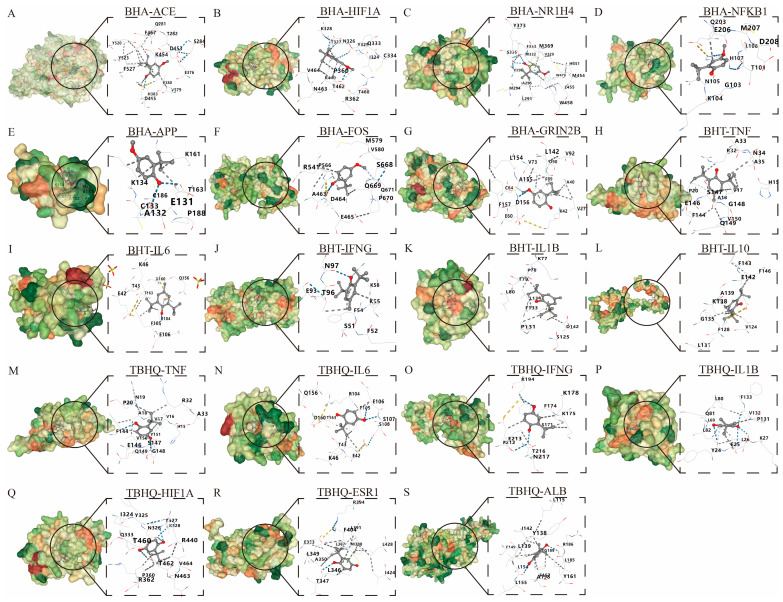
Molecular docking of BHA, BHT, and TBHQ to core targets. The optimal docking results of BHA and ACE (**A**), HIF1A (**B**), HR1H4 (**C**), NFKB1 (**D**), APP (**E**), FOS (**F**), and GRIN2B (**G**). The optimal docking results of BHT and TNF (**H**), IL6 (**I**), IFNG (**J**), IL1B (**K**) and IL10 (**L**). The optimal docking results of TBHQ and TNF (**M**), IL6 (**N**), IFNG (**O**), IL1B (**P**), HIF1A (**Q**), ESR1 (**R**) and ALB (**S**). Receptor proteins are depicted in green, the compound is shown in dark gray, and the dark blue dashed lines represent hydrogen bonds.

A molecular docking analysis was performed on BHA, BHT, and TBHQ (Table 3) using their core targets in hepatotoxicity, nephrotoxicity, and neurotoxicity, respectively. Five docking conformations were generated for each docking. The affinity docking results and the minimum binding free energy are shown in Table 4. An affinity > −4 kcal/mol was defined as a very weak or lack of binding capacity; −7 kcal/mol < affinity < −4 kcal/mol was defined as a moderate binding capacity; and an affinity < −7 kcal/mol was defined as a strong binding capacity. Via energy sorting, conformations with binding free energies lower than −5 kcal/mol were screened, and the conformation with the lowest binding free energy was selected as the target conformation for analysis. All docking results were found to be less than −5 kcal/mol, indicating a moderate binding force, except for those of BHA, which docked to APP at −4.3 kcal/mol (Figure 6).

### 3.6. KEGG Enrichment Analysis of Core Targets

**Figure 7:**
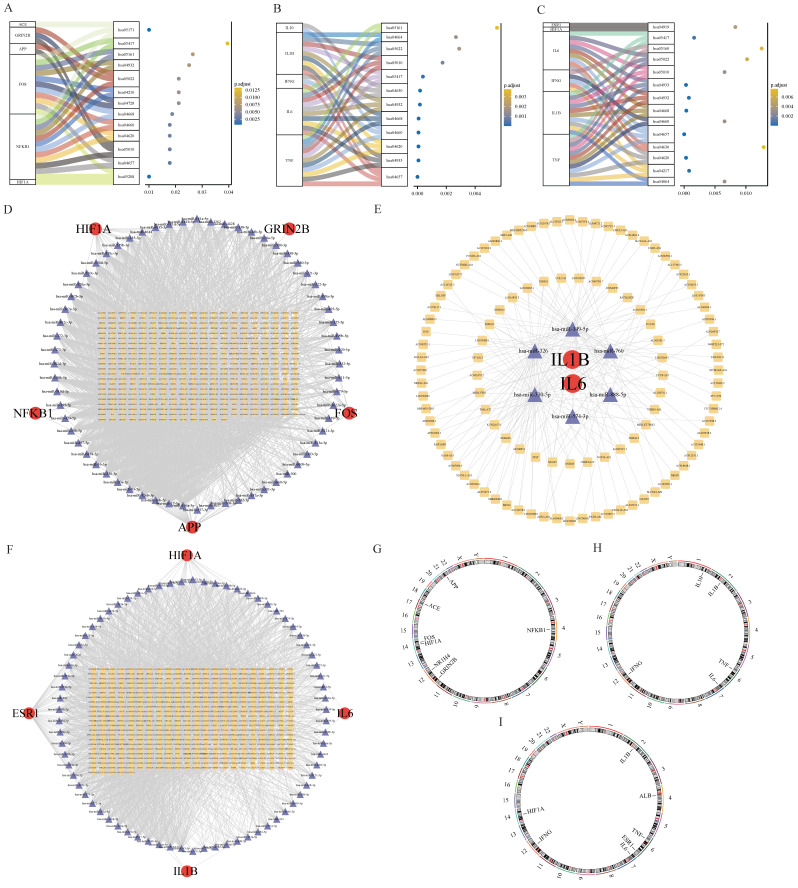
Core targets of BHA (**A**), BHT (**B**), and TBHQ (**C**) in hepatotoxicity, nephrotoxicity, and neurotoxicity and a mulberry bubble diagram of the KEGG enrichment analysis; mRNA-miRNA-IncRNA interaction network of core targets of BHA (**D**), BHT (**E**), and TBHQ (**F**); chromosome position analysis of core targets of BHA (**G**), BHT (**H**), and TBHQ (**I**).

The KEGG analysis revealed that the core targets of BHA’s action in hepatotoxicity, nephrotoxicity, and neurotoxicity were mainly related to the pathways involved in chemical carcinogenesis-reactive oxygen species, IL-17 signaling, Alzheimer’s disease, Toll-like receptor signaling, and T-cell receptor signaling. The core genes of BHT’s action were mainly related to the pathways involved in AGE-RAGE signaling in diabetic complications, Toll-like receptor signaling, T-cell signaling, tumor necrosis factor (TNF) signaling, and NF-κB signaling. The core genes of TBHQ’s action in liver, kidney, and nerve injury were mainly related to the NF-κB signaling, necroptosis, Toll-like signaling, JAK-STAT cell signaling, IL-17 signaling, and T receptor signaling pathways. A mulberry bubble diagram was constructed, and it mainly indicates the relationship between the core genes of BHA, BHT, and TBHQ and the pathways involved in hepatotoxicity, nephrotoxicity, and neurotoxicity (Figure 7A–C). The KEGG pathway analysis identified various molecular mechanisms of BHA, BHT, and TBHQ. BHA was found to primarily target ACE, HIF1A, and NFKB1, modulating the IL-17 and reactive oxygen species (ROS) signaling pathways and contributing to hepatotoxicity, nephrotoxicity, and neurotoxicity. BHT was found to predominantly act through IL1B, IL6, and TNF to influence both the TNF and NF-κB signaling pathways. TBHQ was found to target HIF1A, IL6, IL1B, and TNF, regulating the IL-17 signaling pathway. These findings emphasize both the shared and unique pathways through which these compounds exert their toxic effects.

### 3.7. mRNA-IncmiRNA-RNA Network

The miRNAs common to BHA, BHT, and TBHQ in the three databases were compared, and IncRNAs were predicted. In these databases, 5 mRNAs, 71 miRNAs, and 433 IncRNAs were found in the interaction network of the core targets of BHA in hepatotoxicity, nephrotoxicity, and neurotoxicity, and an mRNA-miRNA-IncRNA interaction network map was drawn in Cytoscape (Figure 7D). In the core target interaction network corresponding to BHT, 2 mRNAs, 6 miRNAs, and 105 IncRNAs were found (Figure 7E); in the core target interaction network corresponding to TBHQ, 4 mRNAs, 74 miRNAs, and 489 IncRNAs were found (Figure 7F).

### 3.8. Chromosome Localization

The human chromosomal locations of the core genes corresponding to the effects of BHA, BHT, and TBHQ were analyzed (Figure 7G–I, respectively). The results showed that the core genes related to the effects of BHA were located on chromosomes 4, 12, 17, and 21; those related to the effects of BHT were located on chromosomes 1, 2, 6, 7, and 12; and those related to the effects of TBHQ were located on chromosomes 2, 4, 6, 7, 12, and 14.

### 3.9. Carcinogenic Risk of Core Targets

**Figure 8:**
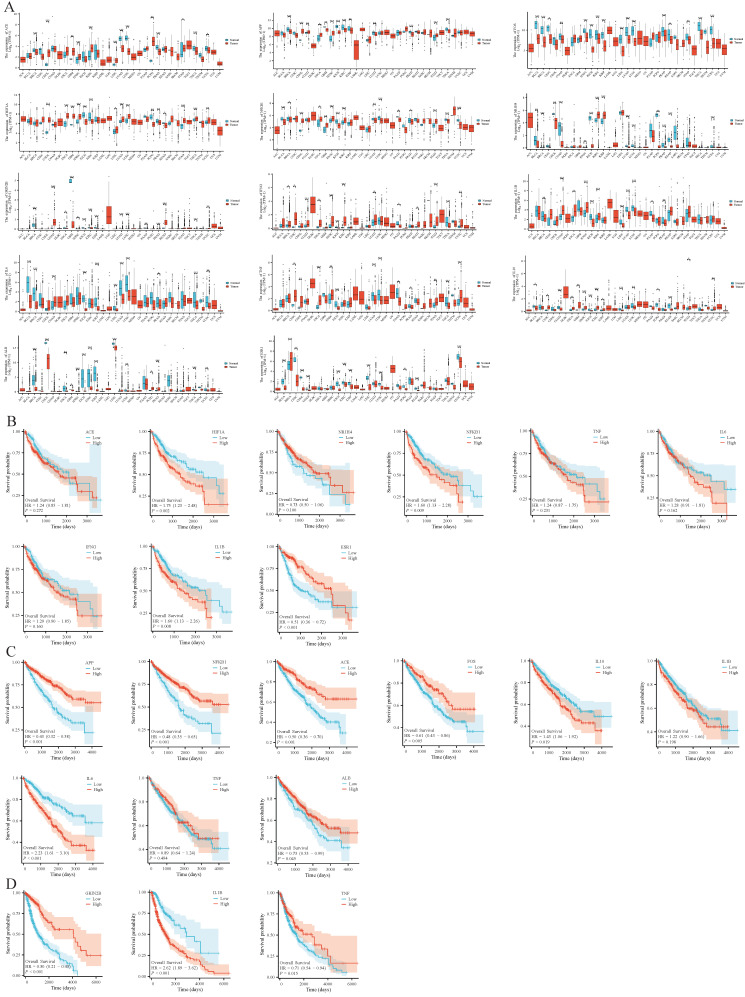
Pan-cancer analysis of core targets of BHA, BHT, and TBHQ, namely, ACE, APP, FOS, HIF1A, NFKB1, NR1H4, GR1N2B, IFNG, IL1B, IL6, TNF, IL10, ALB, and ESR1 (**A**); KM survival analysis of ACE, HIF1A, NR1H4, NFKB1, TNF, IL6, IFNG, IL1B, and ESR1 in liver cancer (**B**); KM survival analysis of C APP, NFKB1, ACE, FOS, IL10, IL1B, IL6, TNF, and ALB in renal clear cell carcinoma (**C**); KM survival analysis of D GRIN2B, IL1B, and TNF in glioma (**D**). * *p* < 0.05, ** *p* < 0.01, *** *p* < 0.001 vs. Normal group.

Using the TCGA database, it was found that the core targets of BHA, BHT, and TBHQ were differentially expressed in breast cancer (BRCA), kidney renal clear cell carcinoma (KIRC), lung adenocarcinoma (LUAD), bladder urothelial carcinoma (BLCA), and uterine corpus endometrial carcinoma (UCEC). Their hepatotoxic, nephrotoxic, and neurotoxic effects may further exacerbate physical diseases and present a carcinogenic risk (Figure 8A). In addition, a Kaplan–Meier (KM) survival analysis was conducted on the core genes in liver cancer, renal clear cell carcinoma, and glioma, and it was found that TNF, IL6, IFNG, and NR1H4 in liver cancer and TNF and IL1B in renal clear cell carcinoma were associated with survival prognosis (Figure 8B–D).

## 4. Discussion

BHA, BHT, and TBHQ are widely used in the food industry and can effectively inhibit the oxidation of fats and oils and maintain the color, fragrance, and taste of foods, especially in fried foods, biscuits, baked foods, and instant foods. However, their safety remains controversial, and the effects of their long-term consumption on human health need to be further evaluated [32]. In the present study, a preliminary toxicity analysis was conducted on BHA, BHT, and TBHQ using the ProTox-3.0, ADMETlab 3.0, and Xundrug databases, and it was found that these compounds may have significant hepatic, renal, and neurotoxic effects. The potential targets of these compounds were successfully identified, in combination with their standard SMILES structural formulas, in the PubChem database, and those relevant to humans were explored through databases such as Swiss TargetPrediction, Batman, SuperPred, and SEA. Subsequently, targets associated with the liver, the kidney, and neurotoxicity were collected from the GeneCards and OMIM databases, and their data were integrated. Then, GO and KEGG enrichment analyses were performed to reveal the key biological processes, molecular functions, and signaling pathways that may be affected by these compounds. A PPI network analysis further identified the core targets, and core genes were screened using Cytohubba. Our molecular docking studies showed that BHA, BHT, and TBHQ have a moderate binding energy with their core targets and may play an important role in toxic effects. The KEGG analysis also revealed pathways associated with chemical carcinogenesis and reactive oxygen species (ROS), as well as multiple signaling pathways, providing a framework for understanding the toxicological effects of these compounds. In addition, the mRNA-miRNA-IncRNA interaction network analysis further revealed potential post-transcriptional regulatory mechanisms. In summary, the comprehensive predictive modeling, target identification, enrichment analysis, and molecular docking studies herein provide multiple insights into the toxic mechanisms of BHA, BHT, and TBHQ, laying the foundation for future health risk assessments and the development of mitigation strategies.

The core genes affected by BHA, BHT, and TBHQ play a potentially important role in liver, kidney, and nerve injury. TNF is prominent because of its critical role in inflammatory responses and cell signaling; it induces the dysfunction of the NF-κB signaling pathway, which leads to chronic inflammation [33]. TNF is a proinflammatory cytokine that has been implicated in various pathological conditions, including liver injury and neurodegenerative diseases. Elevated levels of TNF are associated with hepatotoxicity, where it mediates hepatic inflammation and apoptosis [34]. Conversely, IL-6 plays a multifaceted role in immune regulation and inflammation. IL6 further amplifies the inflammatory response by activating the JAK-STAT signaling pathway [35]. IL6 is involved in the acute-phase response and is associated with renal and neurological injury. Studies have shown that IL6 can exacerbate kidney injury by promoting inflammation and fibrosis [36,37]. In the context of neurotoxicity, IL6 has been implicated in the progression of neurodegenerative diseases, and it is able to influence neuronal survival and function [38]. IL-1B can directly lead to hepatocyte injury by activating apoptotic and necrotic pathways [39], and in some neuropathies, such as stroke and Alzheimer’s disease, the overexpression of IL-1B may promote neuronal apoptosis and lead to neurological dysfunction [40]. HIF1A can promote the expression of inflammatory factors and enhance the inflammatory response, leading to the further development of liver injury [41,42]. The activation of HIF1A promotes hepatocyte survival [43]; however, HIF1A may also promote apoptosis in the setting of severe injury, leading to the further impairment of liver function [44]. NFKB1 is involved in regulating the expression of a variety of genes involved in inflammation, cell proliferation, and survival [45]; its activation is a critical issue in the inflammatory response, and its dysregulation is associated with various diseases, including liver and kidney diseases [46]. ACE catalyzes the conversion of angiotensin I to angiotensin II, a powerful vasoconstrictor that leads to changes in hepatic blood flow and may aggravate liver injury [47]. In general, BHA, BHT, and TBHQ may mediate the biological mechanisms of TNF, IL6, IL1B, HIF1A, NFKB1, and ACE in the liver, kidney, and nerves, resulting in some physical effects, providing a basis for the further study of their mechanisms of action and potential defense.

The KEGG pathway enrichment analysis revealed key signaling pathways associated with the potential hepatotoxicity, nephrotoxicity, and neurotoxicity of BHA, BHT, and TBHQ, such as the IL-17 signaling pathway; this pathway is involved in autoimmune and inflammatory responses, and its dysregulation is associated with a variety of chronic diseases [48]. The IL-17 signaling pathway indirectly triggers the release of proinflammatory cytokines, including TNF and IL-6 [49], which subsequently activate the NF-κB pathway, thereby exacerbating inflammatory liver diseases and related pathological conditions [50]. The activation of IL-17 can lead to an increase in immune cells and the production of inflammatory mediators [51], which may contribute to the nephrotoxic and hepatotoxic effects of BHA, BHT, and TBHQ. In BHT, TBHQ binds to TNF and other factors to activate the TNF signaling pathway, which leads to hepatotoxicity, nephrotoxicity, and neurotoxicity and further leads to the development of cancer [4]. However, the NF-κB signaling pathway emerged as significant for all three compounds. This pathway has been shown to play a role in mediating inflammatory responses and cellular stress, which is critical in the pathogenesis of a variety of diseases, including cancer [52]. The activation of NF-κB can lead to the transcription of proinflammatory cytokines, which may exacerbate tissue, liver, and kidney injury [53]. The JAK-STAT signaling pathway plays a critical role in mediating responses to cytokines and growth factors [54]. BHT exerts its effects by targeting TNF, thereby activating the JAK-STAT signaling pathway, which subsequently promotes immune activation and oxidative stress responses in vivo [55]. The dysregulation of this pathway is associated with a variety of pathological conditions, including cancer and chronic inflammatory diseases [56]. The association of this pathway with genes central to the action of BHA, BHT, and TBHQ suggests that these compounds may influence cell signaling by predisposing individuals to certain impairments. These findings highlight the need for a comprehensive toxicological assessment of BHA, BHT, and TBHQ, particularly regarding their long-term effects on human health. The core genes of BHA, BHT, and TBHQ are involved in pathways that are associated with inflammation, apoptosis, and cell signaling and that play critical roles in the pathogenesis of hepatotoxicity, nephrotoxicity, and neurotoxicity. In particular, the involvement of the NF-κB signaling pathway, among others, suggests a potential mechanism by which these compounds may exert their toxic effects.

In this study, the potential toxicological effects of BHA, BHT, and TBHQ were comprehensively analyzed, revealing important insights into their relationship with liver, kidney, and nerve injury. However, shortcomings remain: First, relying on compound predictions for toxicity assessment, while valuable, may not allow for the complexity of biological interactions or the multifactorial nature of in vivo toxicity to be fully elucidated. The databases used, such as ProTox-3.0 and ADMETlab 3.0, provide a fundamental understanding but may lack the specificity and sensitivity required for accurate predictions in a clinical setting. In addition, this study focused on human data and ignored specific differences. In addition, the use of GO and KEGG analyses without experimental validation limits the reliability of the conclusions drawn. While our computational models provide mechanistic hypotheses for BHA/BHT/TBHQ toxicity, the absence of experimental validation in cellular or animal systems remains a limitation. Future studies integrating multi-omics profiling and targeted in vivo exposure assays are warranted to confirm these predictions. Future studies should further look at practice to validate the potential outcomes of action.

## 5. Conclusions

This study systematically investigates the toxicological profiles and molecular interaction mechanisms of BHA, BHT, and TBHQ, with particular focus on their hepatotoxic, nephrotoxic, and neurotoxic potentials. By employing integrative computational and mechanistic approaches, this research enhances methodological frameworks for food additive risk assessments. The findings propose the need for innovative strategies to redefine risk evaluation paradigms through targeted biomarker discovery coupled with pathway prioritization. To strengthen organ-specific toxicity validations, future studies should incorporate dose–response modeling alongside advanced methodologies such as multi-omics.

## Figures and Tables

**Table 1:** Summary of databases and software.

Database and Software	Database Link and Software Version
Public Chemical Database, Pubchem	https://pubchem.ncbi.nlm.nih.gov/
Swiss Target Prediction	http://www.swisstargetprediction.ch/
Universal Protein Resource, UniProt	https://www.uniprot.org/
GeneCards-The Human Gene Database	https://www.genecards.org/
Protein Data Bank, PDB	http://www.rcsb.org/
Xiantao Academic	https://www.xiantaozi.com/
ProTox 3.0—Prediction of Toxicity of Chemicals, ProTox 3.0	https://tox.charite.de/protox3/index.php?site=home
ADMETlab, ADMETlab 3.0	https://admetlab3.scbdd.com/
Xundrug Database	https://xundrug.cn/
microRNA Database, miRDB	http://mirdb.org
starBase: A Database for MicroRNA-Target Interaction, starBase	https://rnasysu.com/encori/
miRWalk: Database for miRNA Target Prediction and Functional Annotations, miRWalk	http://mirwalk.umm.uni-heidelberg.de
SuperPred	https://prediction.charite.de/
Similarity ensemble approach, SEA	https://sea.bkslab.org/
Bioinformatics Analysis Tool for Molecular mechANism of Traditional Chinese Medicine, BATMAN-TCM	http://bionet.ncpsb.org.cn/batman-tcm/index.php/Home/document/index
Online Mendelian Inheritance in Man, OMIM	https://www.omim.org/
Cavity-detection guided Blind Docking 2, CB-dock2	https://cadd.labshare.cn/cb-dock2/php/index.php
Gencode database	https://www.gencodegenes.org/
Cytoscape	Cytoscape Consortium (San Diego, CA, USA), 3.9.0
RStudio	Public Benefit Corporation (Boston, MA, USA), 4.3.3
Pymol	Schrödinger, Limited Liability Company (New York, NY, USA), 2.6

**Table 2:** List of toxicities of compounds.

No.	Compound Name	Predicted LGC50	Predicted LC50DM	Predicted LC50FM	Predicted LD50	ADI	Maximum Dosage
1	Butylated Hydroxyanisole (BHA)	4.252	5.75	5.083	880 mg/kg	≤1 mg/kg/d	≤200 mg/kg (EFSA)
2	Butylated Hydroxy Toluene (BHT)	3.844	5.293	5.296	650 mg/kg	≤0.25 mg/kg/d	≤200 mg/kg (EFSA)
3	Tert-Butylhydroquinone (TBHQ)	4.524	5.276	4.955	700 mg/kg	≤0.7 mg/kg/d	≤200 mg/kg (FDA)

**Table 3:** List of molecular docking compounds.

No.	PubChem CID	Compound Name	IUPAC Name	Abbreviation	MW	MF	Canonical SMILES	Structure
1	CID 8456	Butylated Hydroxyanisole	2-tert-butyl-4-methoxyphenol	BHA	180.24	C_11_H_16_O_2_	CC(C)(C)C1=C(C=CC(=C1)OC)O	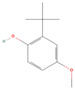
2	CID 31404	Butylated Hydroxy Toluene	2,6-ditert-butyl-4-methylphenol	BHT	220.35	C_15_H_24_O	CC1=CC(=C(C(=C1)C(C)(C)C)O)C(C)(C)C	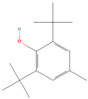
3	CID 16043	Tert-Butylhydroquinone	2-tert-butylbenzene-1,4-diol	TBHQ	166.22	C_10_H_14_O_2_	CC(C)(C)C1=C(C=CC(=C1)O)O	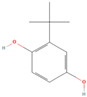

**Table 4:** Summary of affinity energies in molecular docking.

	Compound	Affinity (kcal/mol)		
Target		CID 8456	CID 31404	CID 16043
ACE		−6	--	--
HIF1A		−5.7	--	−5.5
NR1H4		−6.3	--	--
NFKB1		−5	--	--
APP		−4.3	--	--
FOS		−5.7	--	--
GRIN2B		−6.1	--	--
TNF		--	−5.5	−5.2
IL6		--	−5.4	−5.5
IFNG		--	−5.1	−5
IL1B		--	−5.3	−5
IL10		--	−6.7	--
ESR1		--	--	−6
ALB		--	--	−7.3

## Data Availability

The original contributions presented in this study are included in the article/Supplementary Materials. Further inquiries can be directed to the corresponding author.

## Supplementary Materials

The following supporting information can be downloaded at https://www.mdpi.com/article/10.3390/foods14071095/s1, Table S1: Targets in hepatotoxicity, nephrotoxicity, and neurotoxicity; Table S2: Potential targets of BHA, BHT, and TBHQ; Table S3: Targets of BHA, BHT, and TBHQ in hepatotoxicity; Table S4: Targets of BHA, BHT, and TBHQ in nephrotoxicity; Table S5: Targets of BHA, BHT, and TBHQ in neurotoxicity; Table S6: Core targets of BHA, BHT, and TBHQ in hepatotoxicity, nephrotoxicity, and neurotoxicity. Figure S1: Venn diagram of targets of BHA, BHT, and TBHQ in hepatotoxicity, nephrotoxicity and neurotoxicity. BHA and hepatotoxicity (**A**); BHT and hepatotoxicity (**B**); C. TBHQ and hepatotoxicity (**C**); BHA and nephrotoxicity (**D**); BHT and nephrotoxicity (**E**); TBHQ and nephrotoxicity (**F**); BHA and neurotoxicity (**G**); BHT and neurotoxicity (**H**); TBHQ and neurotoxicity (**I**).

## Abbreviations

BHA	butylated hydroxyanisole
BHT	butylated hydroxytoluene
TBHQ	tert-butylhydroquinone
GO	Gene Ontology
KEGG	Kyoto Encyclopedia of Genes and Genomes
IL-17 signaling	interleukin-17 signaling
JAK-STAT signaling	Janus kinase-signal transducer and activator of transcription signaling
NF-κB signaling	nuclear factor kappa-light-chain-enhancer of activated B cells signaling
NAFLD	non-alcoholic fatty liver disease
BP	biological process
MF	molecular function
CC	cellular component
PPI	protein–protein interaction
MCC	maximum clique centrality
MNC	maximum neighborhood centrality
EC	eigenvector centrality
RCSB	Research Collaboratory for Structural Bioinformatics
PDB	Protein Data Bank
MAPK	mitogen-activated protein kinase
PI3K-Akt signaling	phosphoinositide 3-kinase/protein kinase B signaling
VEGF signaling	vascular endothelial growth factor signaling
FoxO signaling	Forkhead Box O signaling
NR1H4	Nuclear Receptor Subfamily 1, Group H, Member 4
ACE	angiotensin-converting enzyme
HIF1A	hypoxia-inducible factor 1-alpha
TNF	tumor necrosis factor
APP	amyloid precursor protein
IL1B	interleukin 1 beta
GRIN2B	Glutamate Receptor, Ionotropic, N-Methyl D-Aspartate 2B
NFKB1	nuclear factor kappa-light-chain-enhancer of activated B cells 1
IL6	interleukin 6
IFNG	interferon gamma
ESR1	estrogen receptor 1
FOS	FBJ osteosarcoma oncogene
IL10	interleukin 10
ALB	albumin
TNF signaling	tumor necrosis factor signaling
KIRC	kidney renal clear cell carcinoma
BRCA	breast cancer
LUAD	lung adenocarcinoma
BLCA	bladder urothelial carcinoma
UCEC	uterine corpus endometrial carcinoma
KM survival	Kaplan–Meier survival
ROS	reactive oxygen species
ERK	extracellular signal-regulated kinase
KEAP1	Kelch-like ECH-associated protein 1
