# Supplementary figures and images for: Exploring the Mechanisms of the Antioxidants BHA, BHT, and TBHQ in Hepatotoxicity, Nephrotoxicity, and Neurotoxicity from the Perspective of Network Toxicology
Source: Foods. 2025 Mar 21;14(7):1095. doi: 10.3390/foods14071095 (PMC11988534; doi:10.3390/foods14071095)

A

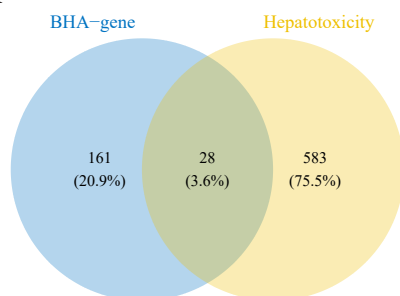

B

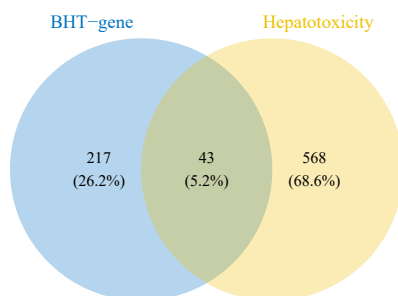

C

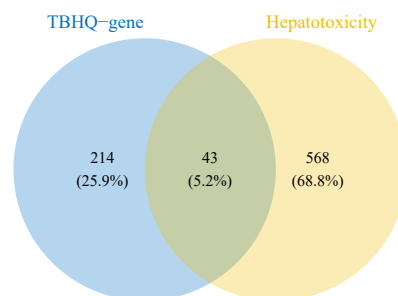

D

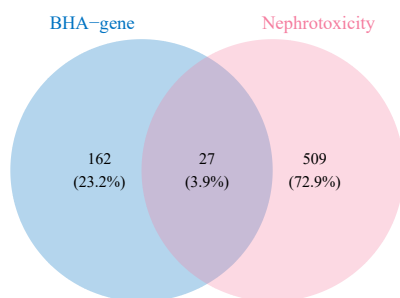

E

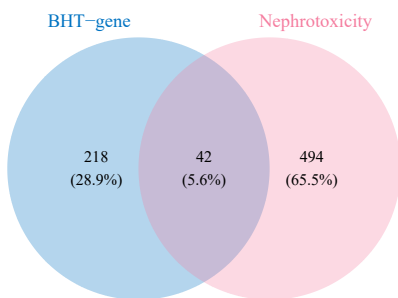

F

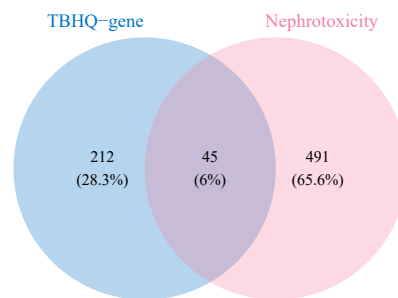

G

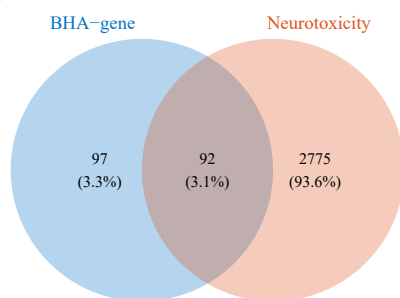

H

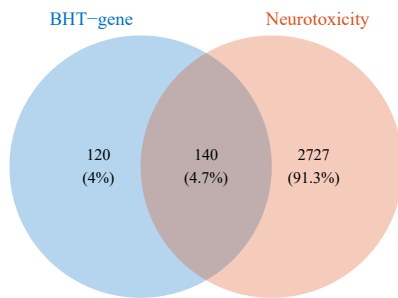

I

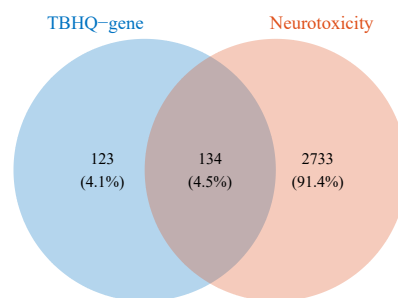

Supplement: Supplementary file 1 [file foods-14-01095-s001.zip › Supplementary Fig S1.pdf]
